# Reversal of oncogene transformation and suppression of tumor growth by the novel IGF1R kinase inhibitor A-928605

**DOI:** 10.1186/1471-2407-9-314

**Published:** 2009-09-04

**Authors:** William N Pappano, Paul M Jung, Jonathan A Meulbroek, Yi-Chun Wang, Robert D Hubbard, Qian Zhang, Meagan M Grudzien, Niru B Soni, Eric F Johnson, George S Sheppard, Cherrie Donawho, Fritz G Buchanan, Steven K Davidsen, Randy L Bell, Jieyi Wang

**Affiliations:** 1 Cancer Research, Global Pharmaceutical Research and Development, Abbott Laboratories, Abbott Park, IL 60064, USA

## Abstract

**Background:**

The insulin-like growth factor (IGF) axis is an important signaling pathway in the growth and survival of many cell and tissue types. This pathway has also been implicated in many aspects of cancer progression from tumorigenesis to metastasis. The multiple roles of IGF signaling in cancer suggest that inhibition of the pathway might yield clinically effective therapeutics.

**Methods:**

We describe A-928605, a novel pyrazolo [3,4-*d*]pyrimidine small molecule inhibitor of the receptor tyrosine kinases (IGF1R and IR) responsible for IGF signal transduction. This compound was first tested for its activity and selectivity via conventional *in vitro *kinome profiling and cellular IGF1R autophosphorylation. Additionally, cellular selectivity and efficacy of A-928605 were analyzed in an IGF1R oncogene-addicted cell line by proliferation, signaling and microarray studies. Finally, *in vivo *efficacy of A-928605 was assessed in the oncogene-addicted cell line and in a neuroblastoma model as a single agent as well as in combination with clinically approved therapeutics targeting EGFR in models of pancreatic and non-small cell lung cancers.

**Results:**

A-928605 is a selective IGF1R inhibitor that is able to abrogate activation of the pathway both *in vitro *and *in vivo*. This novel compound dosed as a single agent is able to produce significant growth inhibition of neuroblastoma xenografts *in vivo*. A-928605 is also able to provide additive effects when used in combination with clinically approved agents directed against EGFR in non-small cell lung and human pancreatic tumor models.

**Conclusion:**

These results suggest that a selective IGF1R inhibitor such as A-928605 may provide a useful clinical therapeutic for IGF pathway affected tumors and warrants further investigation.

## Background

Insulin-like growth factor signaling plays an important role in development and adult homeostasis by supporting growth and survival of multiple cell and tissue types [1]. These important functions are a direct result of the ability of IGF signaling to activate both the anti-apoptotic AKT pathway and the mitogenic extracellular signal regulated kinase (ERK) pathway [1]. However, when this elegantly balanced multi-component signaling system is perturbed, the dual roles of IGF signaling in both survival and proliferation make this pathway a likely contributor to cancer biology. Aberrant IGF signaling has been implicated in multiple aspects of tumor progression including oncogenic transformation, cell proliferation, evasion of apoptosis, tumor cell invasion and metastases [1,2]. Additionally, IGF signaling has been implicated in resistance to multiple current clinical therapeutics [3-8]. These central roles in tumor initiation, growth and progression make the IGF pathway an ideal candidate pathway to target therapeutically.

IGF pathway signal transduction is thought to occur exclusively through extracellular ligand activation of the insulin-like growth factor-1 receptor (IGF1R) and the insulin receptor (IR) [9]. These plasma membrane proteins are members of the receptor tyrosine kinase family and are composed of two extracellular α-subunits disulfide bonded to two single pass membrane spanning β-subunits that contain the cytoplasmic tyrosine kinase activity. The receptors are present either as homodimers or hybrid receptors composed of IGF1R and IR heterodimers, and are activated by binding of the ligands IGF1 and IGF2, as well as insulin when the hybrid receptor is present [10]. The insulin receptor has two alternatively spliced variants known as IR_A _and IR_B_. IR_A _is missing exon 11 which encodes an extra 12 amino acids for the alpha subunit of the IR_B _form of the receptor. IR_B _binds to only insulin while IR_A _is known to associate with both insulin and IGF2 [11]. IGF1 acts both as a circulating hormone and as a tissue growth factor and is expressed in most normal tissues, while IGF2 is predominantly a pre-natal growth hormone in humans [1,12]. Binding of these ligands to the receptors initiates various signaling cascades that ultimately lead to the anti-apoptotic and proliferative signals through the AKT and ERK pathways, respectively. After ligand binding the receptors become autophosphorylated and activate downstream signaling pathways that ultimately lead to proliferation through effects on cell cycle proteins like Cyclin D1 and p27 [13]. In addition, survival is increased, for instance, by phosphorylation of the pro-apoptotic protein Bad by AKT and ERK, which results in its sequestration and inactivation by 14-3-3 [14].

The ligand-dependent activation of IGF signal transduction and numerous pathway players increases the complexity of the role of this pathway in cancer. Multiple studies indicate the presence of increased circulating levels of IGF1 in the plasma of patients with prostate, breast and colon cancers [15]. Several studies also implicate over-expression of IGF2 in colon cancer and in ovarian cancer [15]. This increase in IGF2 expression in colon and ovarian cancer is likely the result of a loss of imprinting as both the maternal and paternal forms of this gene are seen in a significant number of these patients [16,17]. In addition, several studies also suggest that there are a number of tumors that show a higher expression level of IGF1R as well as an increased ratio of IR_A _to IR_B _[15,18,19].

In this study, we report on the *in vitro *and *in vivo *activity of a novel pyrazolo [3,4-*d*]pyrimidine small molecule inhibitor of IGF1R, A-928605. This compound was found to be selective when analyzed in a panel of *in vitro *kinase assays, followed by profiling *in vivo *with a novel IGF1R driven tumor model. Since there are no known activating mutations for IGF1R, we generated a constitutively activated chimeric receptor that is capable of transforming NIH-3T3 cells into a rapidly growing xenograft flank tumor model that is shown to be exquisitely dependent upon IGF signal transduction. The effects of A-928605 in this transformed cell line were directly compared to its effects in the non-transformed control cell line, and resulting data demonstrated that its biological activities are predominantly mechanism-based. The overall positive effects led to analysis of this compound in a patient derived cell line models of neuroblastoma as well as combination studies in non-small cell lung cancer (NSCLC) and pancreatic carcinoma in which the small molecule inhibitor A-928605 was shown to be efficacious.

## Methods

### Small-molecule inhibitors

Lapatinib, gefitinib, dastinib, sorafinib, imatinib and erlotinib were prepared using published synthetic procedures. A-928605 was prepared as previously described [20].

### Molecular biology and cell lines

NIH-3T3, SK-N-FI, MiaPaCa-2 and HCC827 cell lines were obtained from the American Type Culture Collection (ATCC, Manassas, VA). Cell lines were cultured and expanded in media according to ATCC guidelines. CD8-IGF1R was generated by an in-frame fusion of the extracellular and transmembrane domains of the human CD8α coding sequence (amino acids 1-207) followed by a BamHI restriction site to the cytoplasmic domain of human IGF1R (amino acids 959-1368). This product was cloned into the pIRESpuro3 vector (Clontech, Mountain View, CA) via AflII/NotI adaptors. NIH-3T3 CD8-IGF1R and vector control lines were generated by transfection of these constructs with FuGENE 6 (Roche Diagnostics, Basel, Switzerland) followed by selection with 2 μg/ml puromycin. Cells were dilution cloned and surviving colonies were screened for expression of the transgene. Cell clones positive for CD8-IGF1R expression and vector alone transfectants were pooled and remained under puromycin selection for the duration of the study.

### Proliferation and soft agar assays

Cells grown in soft agar were plated at 5,000 cells/well in 12-well tissue culture plates in a top layer of 0.3% noble agar on a 0.7% bottom layer. Media containing the final desired concentration of small molecule inhibitor was used as the agar overlay. For proliferation assays, 5,000 cells per well were plated in 96-well tissue culture treated plates and allowed to attach overnight. The following day small molecule inhibitors or DMSO control were added in duplicate at the indicated concentrations and cells were placed in tissue culture incubators for 72 hours. Cell viability was determined by an MTS assay (CellTiter Aqueous One, Promega, Madison, WI).

### Western blot analyses

Antibodies used included rabbit anti-ERK1/2 pTpY185/187 (Biosource, Camarillo, CA), rabbit anti-IGF-1Rβ and rabbit anti-Cyclin D1 (Santa Cruz Biotechnology, Inc., Santa Cruz, CA), rabbit anti-phospho-IGF-1Rβ (Tyr1135/1136)/IR (Tyr1150/1151), rabbit antiphospho-AKT (S473), rabbit anti-AKT, rabbit anti-ERK1/2, (Cell Signaling Technology, Inc., Beverly, MA) and mouse anti-ACTIN (Sigma, St. Louis, MO). All cell lysate samples were lysed directly in 2× LDS NuPAGE sample buffer with reducing reagent (Invitrogen, Carlsbad, CA) and were analyzed for levels of phosphorylated and total signaling proteins by Western blotting using the NuPAGE electrophoresis and transfer systems (Invitrogen, Carlsbad, CA) and near infrared detection.

### DNA microarray analysis

Cells were treated with 1 μM of compound or DMSO control (0.000002% final concentration) and grown under normal conditions for 24 hours. Cells were then directly lysed in QIAzol RNA lysis reagent (Qiagen, Valencia, CA). Samples were homogenized using an Ultra Turrax T 25 homogenizer (IKA, Wilmington, SC) and then processed with the RNeasy 96 Universal Tissue 8000 kit (Qiagen, Valencia, CA) using a BioRobot 3000 liquid handler (Qiagen, Valencia, CA) and a Sigma 4K15C centrifuge (Sigma, Osterode am Harz, Germany) according to the Qiagen-prescribed protocol. Approximately 2 μg of total RNA for each sample were converted into 20 μg of fragmented, biotin-labeled cRNA with a TargetPrep BioRobot 3000 (Qiagen, Valencia, CA) using a SuperScript Double-Stranded cDNA Synthesis Kit (Invitrogen, Carlsbad, CA), T7-Oligo(dT) primer (Integrated DNA Technologies, Coralville, IA), MessageAmpTM II-Biotin Enhanced In Vitro Transcription Reagents (Ambion, Austin, TX), and 5× Fragmentation Buffer (Qiagen, Valencia, CA). Ten micrograms of fragmented, labeled cRNAs for each sample were hybridized with an Affymetrix Mouse Genome 430A 2.0 array (Affymetrix, Santa Clara, CA) using standard Affymetrix protocols. After hybridization, the chips were washed and stained using Affymetrix GeneChip Fluidics_450 Stations and visualized with the Affymetrix GeneChip Scanner. GeneChip Operating Software (v1.4.036) was used to quantify perfect match and mismatch features on the scanned microarray images. After quantification, quality control was assured using the Affymetrix Power Tools Quality Reporter (v1.0.04) quality control metrics.

Data normalization and analysis were performed using Resolver 7.0 software (Rosetta Inpharmatics, Seattle, WA). Ratios were built for the average of each treatment versus the group average and a p-value was calculated for every-fold change using the Resolver Affymetrix error model [21]. Transcripts and experimental treatments were grouped for visualization by agglomerative hierarchical clustering using the Pearson correlation and the average link in as described in each figure. Probes with a difference of +/- 1.5-fold with a False Discovery Rate (FDR) < 0.05 were considered statistically significant [22]. Transcripts that had statistically significant differences vs. the vehicle control group were investigated through the use of Ingenuity Pathway Analysis (IPA) version 6.5 software (Ingenuity Systems, Redwood City, CA) to identify the most significantly over-represented biological pathways and categories of gene ontology with coordinated gene regulation. Array data has been deposited in the public Gene Expression Omnibus database, accession GSE14024.

### Tumor implantation and anti-tumor efficacy studies

All animal studies were conducted in accordance with the guidelines established by the internal Institutional Animal Care and Use Committees. For CD8 IGF1R flank tumors, 5.0 × 10^5 ^cells/mouse were mixed with an equal volume of Matrigel (BD Biosciences) and injected subcutaneously into the flank of 6- to 8-week-old female nu/nu mice (22 g; Charles River Laboratories). The tumors were allowed to grow approximately 225 mm^3^, and mice were reassigned into treatment groups (N = 10) based on tumor volume. At the initiation of treatment, the mean tumor volume ± SEM of the groups was approximately 231 ± 10 mm^3^. For MiaPaCa-2 flank tumors, 1.0 × 10^6 ^cells/mouse were mixed with an equal volume of Matrigel and injected subcutaneously into the flank of 6- to 8-week-old female SCID mice (20 g; Charles River Laboratories). The tumors were allowed to grow approximately 225 mm^3^, and mice were reassigned into treatment groups (N = 10) based on tumor volume. At the initiation of treatment, the mean tumor volume ± SEM of the groups was approximately 210 ± 30 mm^3^. For HCC-827 flank tumors, a brei was made from HCC-827 tumors maintained by serial passage in mice and injected subcutaneously into the flank of 6- to 8-week-old female SCID mice (20 g; Charles River Laboratories). The tumors were allowed to grow approximately 225 mm^3^, and mice were reassigned into treatment groups (N = 10) based on tumor volume. At the initiation of treatment, the mean tumor volume ± SEM of the groups was approximately 300 ± 20 mm^3^. In all flank models, tumor growth was periodically analyzed by measurement with digital calipers and tumor volume was estimated from the formula (*L *× *W *^2^)/2. For all flank models, the effect of treatment on tumor growth rate was assessed by determining %T/C [(mean tumor volume of treated group on day X ÷ mean tumor volume of control group on day X) × 100].

**Figure 1:**
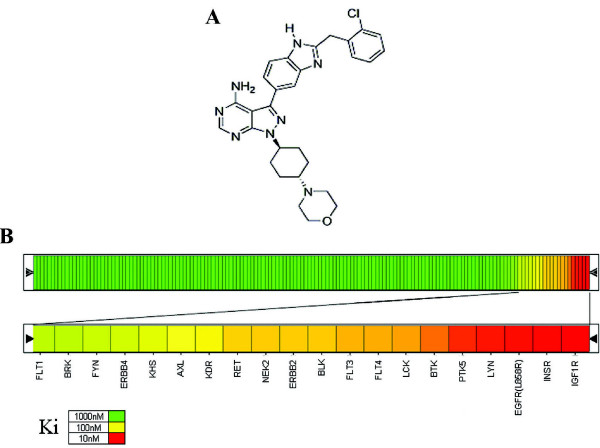
**Chemical structure and activity of A-928605**. **A**, The chemical name of A-928605 is 3-(2-(2-chlorobenzyl)-1 *H*-benzo [d]imidazol-5-yl)-1-((1 *r*, 4 *r*)-4-morpholinocyclohexyl)-1 *H*-pyrazolo [3,4-*d*]pyrimidin-4-amine. **B**, Kinome profile analysis of A-928605 on 156 unique kinases. Heat map colors correspond to the relative Ki against each kinase, where Ki greater than 1,000 nM are colored green, near 100 nM are yellow and Ki of roughly 10 nM are shaded red.

For SK-N-FI renal subcapsule tumors, mice were anesthetized with intramuscular injections of ketamine (40 mg/kg) and rompum (5 mg/kg) before surgery. A small incision was made along the right flank of the mouse and 0.01 ml of 2 × 10^6 ^SK-N-FI cells were mixed with an equal volume of Matrigel and injected just under the subcapsule of the exposed kidney of 10-12 week old male SCID mice (25 g; Charles River Labs). The peritoneal cavity was closed with 4-0 suture and skin incision was closed with clip. Fourteen days after tumor cell injection, tumor growth was assessed in cohort mice by necropsy (see Figure 1B) and study mice were reassigned at that time to treatment groups (N = 10) based on injection order. At the end of the dosing period, tumor weight was determined by comparing the weight of the tumor bearing kidney to that of the contralateral kidney. The effect of treatment on tumor growth was assessed by determining %T/C [(mean tumor weight of treated group on day X ÷ mean tumor weight of control group on day X) × 100].

For all animal studies, administration of therapeutic compounds are described in the figure legends.

### Statistical analysis

Differences between specified groups were analyzed using the Student's *t *test (two-tailed) for comparing two groups, with *P *< 0.05 considered statistically significant.

## Results

### A-928605 is a potent and selective kinase inhibitor of IGF1R

**Figure 2:**
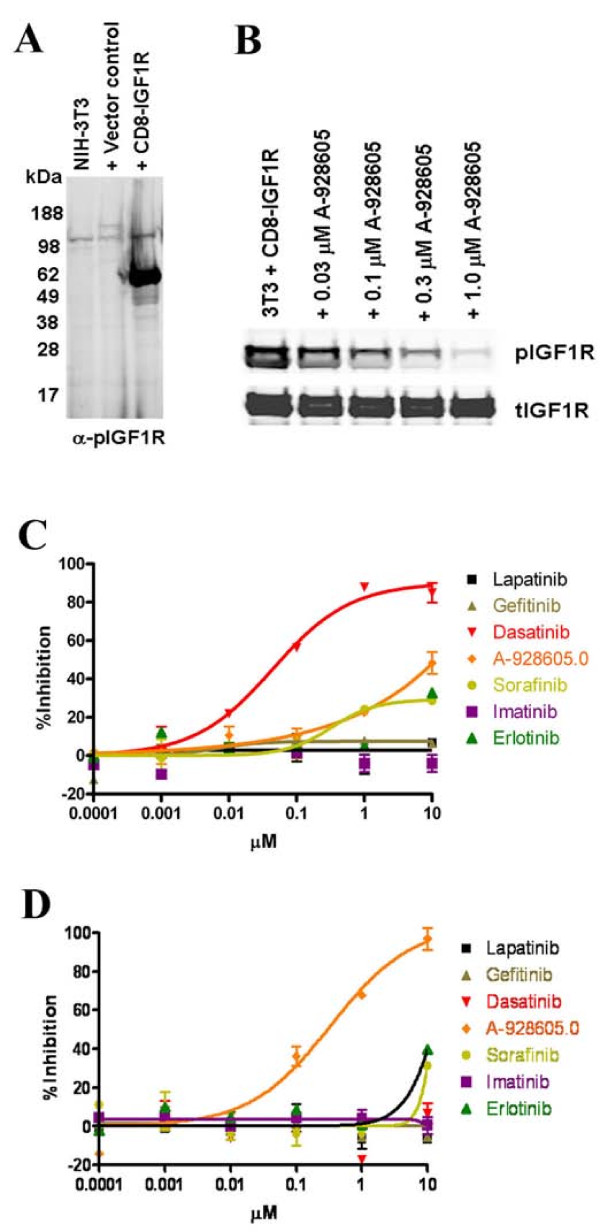
**A-928605 is a potent inhibitor of IGF signal transduction as seen in a model transformed cell line.A**, Parental NIH 3T3 cells compared to the vector control and CD8-IGF1R lines show a strong pIGF1R signal in the CD8-IGF1R line. All cells were grown in normal growth media containing 10% FBS. **B**, Cells were treated for 1 hour with A-928605 in a dose response analysis as shown. The CD8-IGF1R fusion protein runs as a doublet [25]. **C and D**, Three day proliferation assay in the NIH-3T3 vector control cells (**C**) or CD8-IGF1R cells (**D**) with increasing amounts of six clinical small molecule inhibitors and A-928605. Compounds were run in duplicate at each indicated concentration. Results are representative of 5 separate assays. Intended targets of each small molecule inhibitor are as follows: lapatinib, ERBB2 and EGFR; gefitinib, EGFR; dasatinib, ABL and SRC; Sorafinib, multi-targeted; imatinib, ABL and KIT; erlotinib, EGFR.

We originally recognized that the pyrazolopyrimidine class of kinase inhibitors possessed moderate potency for IGF1R during a high throughput screen of our compound repository [20]. A-928605 (Figure 2A) is an optimized pyrazolopyrimidine that has previously been reported to have an IGF1R *in vitro *enzymatic IC_50 _of 37 nM as well as an IC_50 _value of 90 nM for inhibition of cellular IGF1R phosphorylation induced by IGF1 in A431 tumor cells [20]. This small molecule inhibitor was tested against an *in vitro *panel of 156 kinases for potential enzyme inhibition and was found to have a Ki of less than 10 nM in only 5 of those kinases (Figure 2B). Two of these five kinases, IGF1R and the closely related IR, were desired targets and confirmed by cellular analysis. However, the three other kinase activities with Ki values below 10 nM could not be confirmed in cellular assays. For example, A-928605 has an IC_50 _value for inhibition of A-431 and HCC827 cellular epidermal growth factor receptor (EGFR) phosphorylation of > 10 μM.

### A-928605 reverses IGF1R-dependent transformation

**Figure 3:**
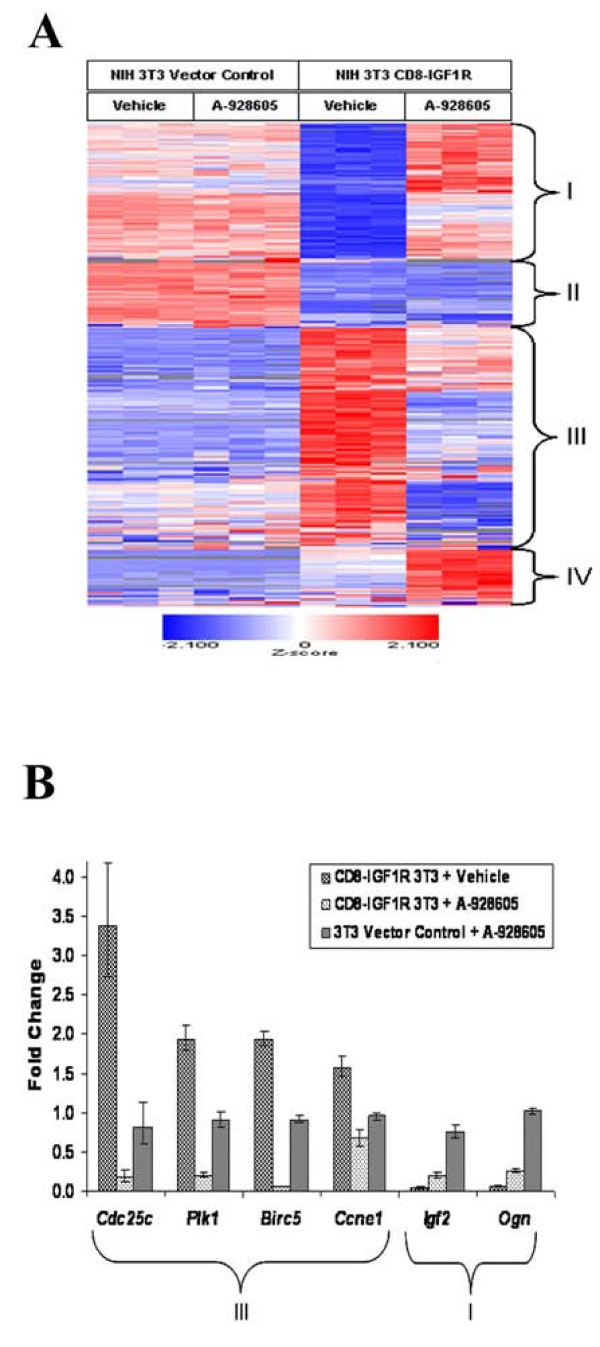
**A composite transcription signature is induced by CD8-IGF1R over-expression and A-928605 treatment of the transformed cellsA**, Expression profile of 1860 Affymetrix probe sets that were differentially expressed between CD8-IGF1R and vector control cells and between CD8-IGF1R vehicle and A-928605-treated cells by at least 1.5-fold and with a 5% FDR. Three independent plates, each represented by a column on the heat map, were analyzed from the vehicle-treated vector control, A-928605-treated vector control, vehicle-treated CD8-IGF1R, A-928605-treated CD8-IGF1R cell groups. Genes exhibiting decreased expression in CD8-IGF1R relative to vector control cells represent classes I (579 transcripts) and II (162 transcripts). Class III (968) and IV (151) transcripts exhibited increased expression in CD8-IGF1R relative to vector control cells. **B**, Relative expression level of key class I and III genes for each treatment group relative to vehicle-treated vector control cells. All vehicle treated vector control cells have gene expression normalized to 1.

The multiple members of the IGF axis, the lack of known receptor activating mutations and the ligand-dependent nature of IGF signal transduction make this pathway difficult to model both *in vitro *and *in vivo*. To bypass these inherent complications, we utilized a previously published technique where fusion of the extracellular sequence of the human T-cell antigen CD8α to the cytoplasmic domain of receptor tyrosine kinases leads to constitutively active receptor kinases that can act as oncogenes [23-25]. CD8α extracellular peptides are known to form dimers [26], and when fused to the cytoplasmic tail of a receptor tyrosine kinase this dimerization results in the kinase domains being brought into proximity and allows constitutive activation. NIH-3T3 cells with over-expressed CD8-IGF1R were generated and the resulting cell line has a strongly constitutively phosphorylated IGF1R cytotail (Figure 3A). After transfection and selection, the surviving CD8-IGF1R positive cells had morphology suggestive of oncogenic transformation by the chimeric protein (see Additional File 1: Supplemental Figure S1A). Treatment of the CD8-IGF1R cell line with increasing amounts of A-928605 produced a dose-dependent inhibition of IGF1R phosphorylation near its known cellular phosphorylation IC_50 _value of ~100 nM (Figure 3B). So while the CD8-IGF1R chimeric kinase is constitutively activated, this activation can be effectively reversed by intervention of the ATP-competitive inhibitor A-928605. This can also be seen at the morphological level as increasing concentrations of A-928605 in the CD8-IGF1R cells results in a reversal of their transformed phenotype (see Additional File 1: Supplemental Figure S1B). A-928605 and six current clinical therapeutic kinase inhibitors were subsequently tested for their ability to inhibit cellular proliferation in the NIH-3T3 CD8-IG1R and vector control cell lines. While there is little effect of A-928605 in the vector control cell line at concentrations sufficient for the inhibition of cellular IGF1R (Figure 3C), it is a potent inhibitor of cellular proliferation of the CD8-IGF1R cells with an IC_50 _of less than 0.3 μM (Figure 3D), indicating that these transformed cells now rely on IGF1R signaling to proliferate. It is interesting to note the switch in signaling dependence between the vector control and CD8-IGF1R lines. Dasatinib (ABL and SRC inhibitor) is a potent inhibitor of the vector control NIH-3T3 cells, but has no effect on the CD8-IGF1R line at concentrations up to 10 μM and none of the other known kinase inhibitors tested showed anti-proliferative activities towards the transformed cells at concentrations below 10 μM. A-928605 is also capable of completely inhibiting the anchorage-independent growth of the transformed NIH-3T3 CD8-IGF1R line when grown in soft agar (see Additional File 2: Supplemental Figure S2). In total, these data suggest that A-928605 can effectively inhibit cellular IGF1R kinase activity and reverse the effects of IGF1R-dependent transformation.

### Expression signature of IGF1R transformation is selectively abrogated by A-928605

**Figure 4:**
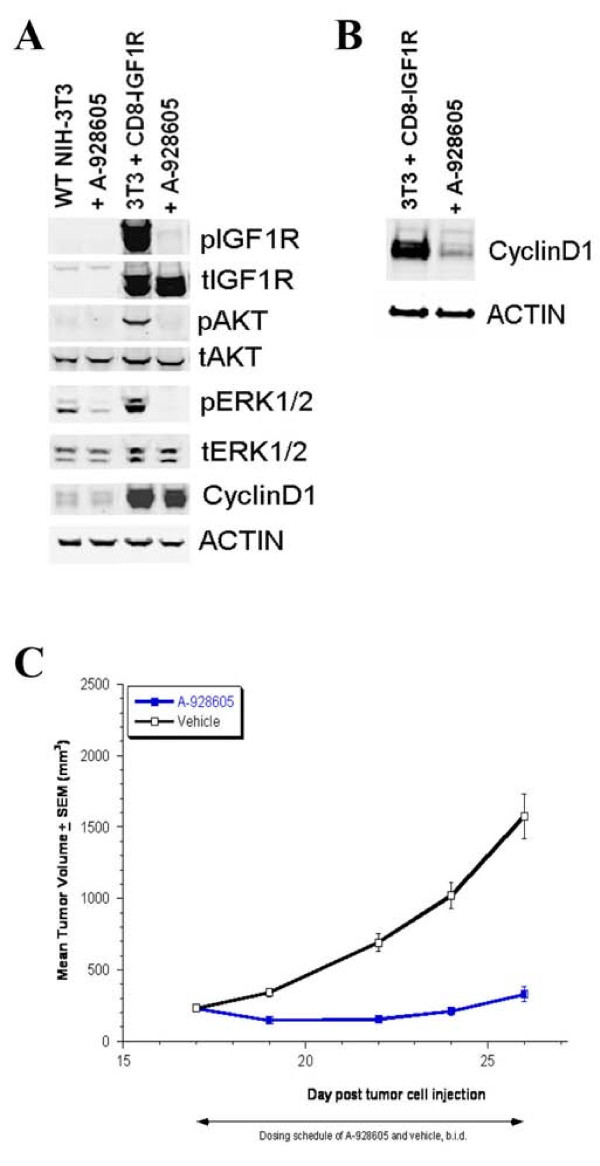
**A-928605 abrogates modifications of IGF pathway signaling proteins in vitro and is efficacious in the CD8-IGF1R xenograft flank model in vivo.** A, One hour of 1 mM A-928605 treatment in vector control and CD8-IGF1R cells results in inhibition of phosphorylation of the IGF1R cytotail and the immediate downstream pathway effectors AKT and ERK1/2. Cyclin D1 is a transcriptional target of the IGF signaling pathway and is therefore not affected by this one hour treatment. B, 24-hours of 1 mM A-928605 treatment in vector control and CD8-IGF1R cells results in a significant decrease in Cyclin D1 expression as seen here and in the microarray results (Figure 3). C, Beginning on day 17 post tumor cell injection, mice were dosed intraperitoneally with A-928605 at 50 mg/kg, twice daily (b.i.d)., or its vehicle for 10 days. At the end of the dosing schedule, the %T/C (A-928605 vs. vehicle) was 21 (P < 0.00001).

Microarray analysis was performed to assess the possible molecular mechanisms underlying the IGF pathway-dependent transformation as well as the effects of IGF axis inhibition by A-928605 in the NIH-3T3 cell lines. Cells were treated with 1 μM of compound or DMSO control and grown under normal conditions for 24 hours. RNA was isolated, and the microarray profiles were analyzed for differential gene expression at a 5% False Discovery Rate (FDR) [22]. A comparison of the vehicle-treated CD8-IGF1R line with the vehicle-treated NIH-3T3 vector control line revealed large gene expression changes including 5,968 transcripts that showed significant changes (5% FDR, fold change > 1.5) split evenly between up- and down-regulation, of which approximately 2,931 were up regulated and 3,037 were down-regulated (Figure 4A). Ingenuity pathway analysis (IPA) of these genes indicated that the constitutive IGF signaling leads to an up-regulation in both mitogenic and anti-apoptotic pathways at the transcriptional level. There is also a significant quantitative increase in connective tissue and extracellular-matrix messenger RNAs including *Tgfβ1 *(2.4-fold) and *Vegfa *(4.3-fold), aiding in the ability of the cells to grow anchorage independently and as tumors (vide infra). As a result of the constitutive activation of the IGF axis, the mRNAs for the ligand *Igf2*, the native receptor *Igf1r*, and IGF binding proteins *Igfbp5 *and *Igfbp7 *were down-regulated, while *Insig2*, *Igfbp4 *and *Igfbp6 *were up-regulated, suggesting an attempt at a compensatory down-regulation of the IGF axis by the cell (see Additional File 3: Supplemental Table 1). Several transcriptional changes in the insulin/IGF pathway observed in these CD8-IGF1R positive cells, such as decreases in the insulin receptor substrate proteins, *Irs1 *and *Irs2*, have been reported for cells treated with IGF1 [27].

The dramatic transcriptional changes induced by the constitutive activation of IGF signaling in the CD8-IGF1R line were largely reversed by treatment with A-928605 (Figure 4A). Of the 4,585 transcripts significantly changed in the A-928605-treated CD8-IGF1R cells relative to vehicle-treated CD8-IGF1R cells, 61% were down-regulated and 1860 transcripts overall were in common with those 5,968 transcripts regulated by CD8-IGF1R expression (Figure 4A). By hierarchical clustering, these genes separated into four major clusters. Classes I and II genes exhibited decreased expression in CD8-IGF1R cells relative to NIH-3T3 vector control cells. Class III and IV genes exhibited increased expression in CD8-IGF1R relative to vector control cells. Expression in class I and III genes were normalized by A-928605 treatment. The transcripts in class III include genes related to the cell cycle and to cell growth and proliferation, which was consistent with the status of IGF signaling as potently mitogenic. Data analysis using IPA software revealed regulation of the G1/S and G2/M canonical pathways in both the CD8-IGF1R resting and treated cells. Indeed, mRNA levels of numerous Cyclins such as *Ccne1*, cell-division cycle proteins including *Cdc25c*, and other genes related to cell proliferation like *Plk1*, were up-regulated by CD8-IGF1R expression and down-regulated upon treatment of the CD8-IGF1R positive cells by A-928605 (Figure 4B). Many of these genes have previously been shown to be up-regulated by IGF1 treatment of MCF-7 cells or down-regulated by IGF1R-antibody treatment of SK-N-AS xenograft tumors [27-29]. Other key genes regulated in those studies, such as *Aurka*, *Birc5*, *Dusp4*, and *Dusp6 *were similarly regulated here (Supplementary Table 1 and Figure 4B). Numerous other growth factor signaling pathways besides the IGF axis were also affected. For example, a variety of cytokines, epidermal growth factors, neurotrophic growth factors, and growth factor receptors were significantly up-regulated by CD8-IGF1R expression and down-regulated upon A-928605 treatment (Supplementary Table 1). In contrast, several important growth factors, including *Igf2 *and *Ogn*, the chemokines *Cxcl5 *and 12, and other growth factor receptors had the opposite expression profile and were in class I. The genes in class IV were up-regulated in CD8-IGF1R cells relative to vector control cells and further up-regulated by A-928605 treatment. Intriguingly, little overall effect on transcription was seen in the vector control line when treated for 24 hours with A-928605, with no statistically significant changes greater than 6-fold and only ten transcripts significantly regulated at all. (Figure 4A, Supplementary Table 1). This almost negligible effect on gene expression in the vector control line combined with the *in vitro *kinase panel and the weak anti-proliferative effect shown in the vector control line in Figure 3C further demonstrate the biological specificity of A-928605 in blocking the IGF axis.

### Translational effects of A-928605 treatment on transformed versus control cells

**Figure 5:**
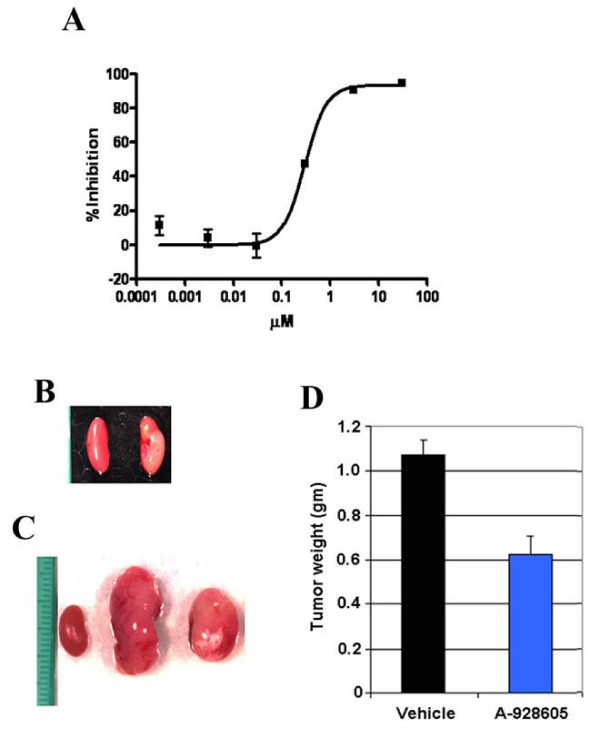
**Efficacy of A-928605 in the SK-N-FI neuroblastoma xenograft as a renal subcapsule model**. **A**, A-928605 inhibits proliferation of SK-N-FI cells *in vitro*. **B-D**, Beginning on day 14 post tumor cell injection, mice were dosed intraperitoneally with A-928605 at 37.5 mg/kg, b.i.d. for 14 days. **B**, Representative photograph taken of the tumor bearing and contralateral kidneys prior to the initiation of dosing on day 14 post tumor cell injection, indicating a tumor weight of 0.07 g. **C**, Representative photograph taken of the tumor bearing and contralateral kidneys at the end of the dosing schedule (day 28). **D**, At the end of the dosing schedule, the %T/C (A-928605 vs. vehicle) was 58 (*P *< 0.001).

The constitutively activated IGF signaling in the CD8-IGF1R line results in phosphorylation of various signaling proteins, including AKT and ERK1/2, that lead to changes in proliferation via cell cycle control by up-regulation and stabilization of proteins like Cyclin D1 (Figure 5A). As expected, treatment of CD8-IGF1R cells with A-928605 leads to inhibition of the phosphorylation of the IGF1R cytotail and downstream effectors AKT and ERK1/2 shortly after treatment (Figure 5A). The microarray analyses shown in Figure 4 were performed on samples after 24 hours of A-928695 treatment to assess the transcriptional changes that occur as a direct result of this pathway inhibition seen in Figure 5A. The results of this analysis point towards gene changes that lead to the significant inhibition of proliferation in the CD8-IGF1R cells versus the vector control cells. One example is the 4.8-fold decrease in *Ccn d1 *transcripts that occurs after 24 hours of A-928605 treatment that is also visible at the translational level (Figure 5B). The data collected at both the transcriptional and translational levels in CD8-IGF1R transformed cells treated with A-928605 provide evidence that this small molecule is effective at inhibiting both the immediate modifications to the IGF signaling pathway as well as its functional read-out in a highly specific manner.

### A-928605 inhibits the growth of IGF1R driven tumors *in vivo*

CD8-IGF1R transformed 3T3 cells readily form xenograft tumors when injected into the flanks of immunocompromised mice (Figure 5C), while the NIH-3T3 vector control cells are not capable of forming growing tumors when injected into host animals (data not shown). We next assessed the *in vivo *anti-tumor efficacy of A-928605 in these CD8-IGF1R tumors. CD8-IGF1R tumors were grown to 225 mm^3 ^and then treated with the compound or vehicle control for 10 days. A-928605 therapy was highly effective at inhibiting growth of this tumor model and resulted in an overall treated versus control (T/C) of 21% at the end of dosing on day 26 of the study (Figure 5C). It is interesting to note the efficiency of this model as tumors can be size-matched at 225 mm^3 ^in roughly 2 weeks post-injection and compound efficacy can be determined in as little as three to four days after initiation of dosing.

**Figure 6:**
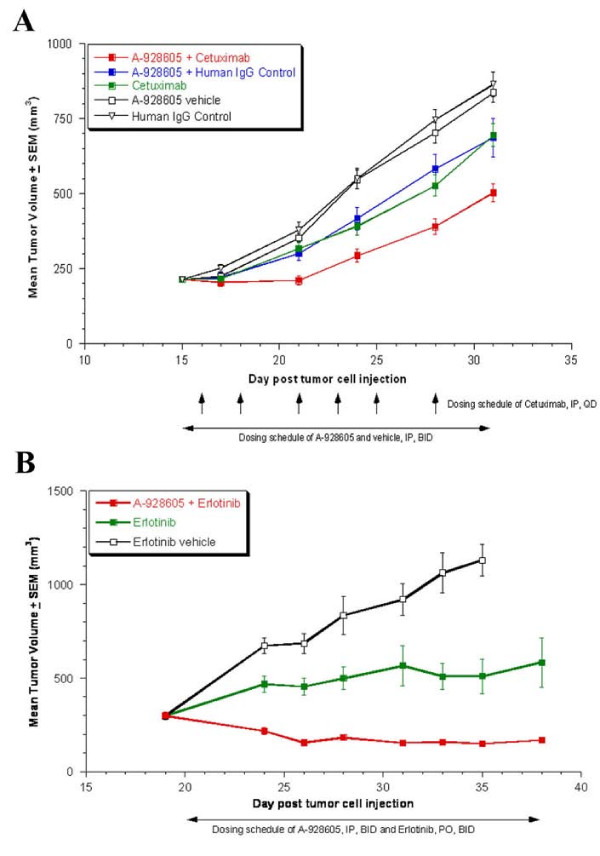
**A, Efficacy of A-928605 and cetuximab, alone and in combination, in the MiaPaCa-2 pancreatic xenograft flank model**. Beginning on day 16 post tumor cell injection, mice were dosed intraperitoneally with A-928605 at 37.5 mg/kg, b.i.d. for 20 days. Cetuximab and human IgG Control were dosed intraperitoneally at 30 mg/kg, once daily, 3×/week for a total of 6 doses. At the end of the dosing schedule, the %T/Cs (combination vs. vehicle and vs. human IgG control) were approximately 60 (*P *< 0.00001) and (combination vs. A-928605 + human IgG Control and vs. cetuximab) were approximately 72 (*P *< 0.05). **B**, Efficacy of A-928605 and erlotinib in combination in the HCC-827 NCSLC xenograft flank model. Beginning on day 20 post tumor cell injection, mice were dosed intraperitoneally with A-928605 at 37.5 mg/kg, b.i.d. for 16 days. Erlotinib was dosed orally at 3.1 mg/kg, b.i.d. for 16 days. At the end of the dosing schedule, the %T/Cs (combination vs. vehicle) was 17 (*P *< 0.00001) and (combination vs. erlotinib) was 27 (*P *< 0.005). In a separate arm of the study, A-928605 as a monotherapy was not consistently statistically different from its vehicle control (data not shown).

### A-928605 is effective as a single agent therapy in a human neuroblastoma renal subcapsule model and in combination with other agents in pancreatic and NSCLC models

Tumor growth inhibition studies were also done utilizing the human neuroblastoma cell line SK-N-FI, the human pancreatic carcinoma line MiaPaCa-2 and the human NSCLC line HCC827 to assess the activity of A-928605 in non-engineered models. The SK-N-FI tumor analysis was performed as autocrine IGF signaling has been associated with the proliferation of neuroblastoma as well as other childhood tumors [30,31]. Accordingly, A-928605 was found to effectively inhibit proliferation of the SK-N-FI cell line *in vitro *(Figure 1A). The *in vivo *experiment was performed by surgical renal subcapsule injection of SK-N-FI cells onto the right kidney capsule of experimental animals (Figure 1B). To assess the anti-tumor efficacy of A-928605, mice were randomized on day 14 post SK-N-FI cell injection, when tumor weights were approximately 0.07 g, as determined from cohort mice culled from the study at that time. Treatment for 14 days with A-928605 (b.i.d., i.p.) resulted in a %T/C of 58 (p < 0.001) (Figure 1C &1D).

The ability of A-928605 to significantly inhibit growth in the SK-N-FI model as a single agent led to the analysis of this agent in other human tumor models in combination with clinically approved therapeutics targeting EGFR. The *in vivo *activity of A-928605 was first assessed in combination with cetuximab, a clinically approved EGFR monoclonal antibody, in the human pancreatic line MiaPaCa-2. First, treatment mice bearing MiaPaCa-2 tumors with A-928605 and cetuximab, alone and in combination, resulted in significant monotherapy activity for each compound, with %T/Cs of approximately 80 (P < 0.05) compared to appropriate controls (Figure 6A). For combination therapy with A-928605 and cetuximab, an additive effect was seen, with the %T/Cs of approximately 72 (P < 0.05) when comparing the combination to each monotherapy and %T/Cs of approximately 60 (P < 0.00001) when comparing the combination to the appropriate negative controls (Figure 6A). The *in vivo *activity of A-928605 was also assessed in combination with erlotinib, a clinically approved small molecule tyrosine kinase inhibitor of EGFR, in the human NSCLC line HCC827. The HCC827 cell line has greatly increased copy number of the EGFR gene (> 20) and harbors an activating mutation caused by a deletion within exon 19 of the EGFR coding sequence [32]. Treatment of size-matched HCC827 xenografts with A-928605 (data not shown) or erlotinib results in partial responses within these study groups, but the combination of these two therapies leads to a synergistic response and regression of the tumors (Figure 6B). These *in vivo *results suggest that A-928605 can be an effective therapeutic as a single agent in IGF-dependent tumors, and it can also be quite effective in combination therapy in tumors known to be sensitive to other targeting agents.

## Discussion

The approach of inhibiting IGF1R with a small molecule is validated by the numerous receptor tyrosine kinases (RTKs) that have been effectively targeted in the treatment of cancer, including Bcr-Abl (imatinib), EGFR (erlotinib, gefitinib) and EGFR/ErbB2 (lapatinib). The success of these small molecule inhibitors in the clinic suggests that numerous other RTKs can be exploited for both their roles in oncogenesis and their relative druggability. The IGF signaling pathway is a multi-component system that plays important roles in both mitogenesis and apoptosis that can ultimately lead to tumorigenesis. The main transducers of the IGF pathway are the RTKs IGF1R and IR. One necessary consideration for targeting the IGF pathway are potential effects on glucose homeostasis in generation of an ATP-competitive inhibitor to IGF1R because of the homology that exists between IGF1R and IR kinase domains (84% similar) and their identical ATP binding sites [33,34]. Indeed, we find that all IGF1R inhibitors that we have generated, including A-928605, have roughly equipotent IC_50_s against IGF1R and IR for both *in vitro *enzyme activity and cellular phosphorylation data (data not shown). This activity is potentially related to increases in resting glucose levels in animal models and patients that is seen after dosing of an IGF1R therapeutic [35]. Despite these complications, the promising efficacy thus far of these treatments in the clinic and the importance of the IGF pathway to human cancer biology warrant additional therapies to go forward into cancer patients where aberrant IGF signaling may be implicated.

In this study we have shown the *in vitro *and *in vivo *anti-proliferative effects of a novel pyrazolo [3,4-*d*]pyrimidine IGF1R small molecule inhibitor. We have characterized A-928605 in an *in vitro *kinase panel and an oncogene-dependent model cell line that enables rapid analysis of the efficacy of IGF1R small molecule inhibitors both *in vitro *and *in vivo*. Using the CD8-IGF1R cell line as a screening tool, A-928605 was shown to be a potent inhibitor of IGF-dependent proliferation. Because of the rapid growth rate and oncogene dependence of the CD8-IGF1R tumor model, the pharmacodynamic and pharmacokinetic properties of A-928605 were ascertained in a tumor setting in less than a week of compound dosing. Additionally, the existence of the isogenic parental vector control cell line served a function in assessing the general non-specific effects of the compound by comparing outcomes in these two cell lines. For instance, A-928605 produced significant changes both by inhibiting early activation of molecules within the IGF signaling pathway and translationally that can be directly attributed to inhibition of IGF1R because none of these effects occurred in the vector control line. A-928605 appears to have few off-target activities in both biochemical and biological contexts when using a kinase panel and microarray analysis of NIH-3T3 cells as rough determinants, respectively. We believe that this cellular analysis is a step beyond and corroborates with the traditional kinome profiling data because it provides an unbiased read-out of the biological consequences of treatment with a small molecule inhibitor in live cells. For example, genes from the microarray analysis include *Cdkn1a *(p21), which when up-regulated by 1.5-fold in A-928605-treated CD8-IGF1R cells could serve to halt the cell cycle in G1 phase. This may lead to the inhibition of proliferation seen upon treatment with A-928605 both *in vitro *and *in vivo*, especially when coupled with the concurrent down-regulation of Cyclin D1 observed at both the transcriptional and protein levels.

It is also interesting to note that activation of the IGF signaling pathway leads to a down-regulation of endogenous IGF pathway components when comparing the untreated vector control line to the CD8-IGF1R line in the microarray experiment. We are not aware of a precedent for this observation and presume this is due to a regulatory feedback loop by the cells in an attempt to quell the high levels of IGF pathway activation observed. This data corroborates well with data currently seen in the clinic for antibodies directed against IGF1R in which the body attempts to compensate for a loss in IGF signaling by increasing levels of circulating IGF1 and Growth Hormone [35] in a potentially related regulatory feedback loop.

The IGF1R inhibitor A-928605 was also able to significantly retard the growth in a model of neuroblastoma. Neuroblastoma is the most common extracranial solid tumor in children, comprising between 8 and 10% of all childhood cancers. Unfortunately, this disease accounts for 15% of all childhood cancer deaths, indicating the poor prognosis of many of the patients [36]. Autocrine IGF signaling has long been recognized as a mitogenic signal for the proliferation of neuroblastoma as well as other childhood tumors [30,31]. Therefore, we analyzed the effects of A-928605 in a surgical model of neuroblastoma using SK-N-FI cells injected into the renal subcapsule. SK-N-FI cells are commonly grown as flank model xenografts, but we believe that the implantation used in this study more accurately reflects the true biology of neuroblastoma by placing the tumor near the adrenal glands of the mouse sympathetic nervous system. This combination of the implantation and waiting two weeks after surgery before treatment with the inhibitor provides a much higher hurdle to assess drug efficacy. Treatment of this preclinical tumor model with A-928605 results in significant growth inhibition suggesting that this compound may be clinically effective at treating childhood tumors like neuroblastoma as well as other adult IGF-dependent tumors.

A-928605 is also efficacious in tumor models when used in combination with clinically approved therapeutics designed against EGFR. For instance, human pancreatic xenografts treated with a combination of the EGFR monoclonal antibody cetuximab and A-928605 results in an additive effect on tumor growth inhibition when compared to either of these agents dosed as monotherapies. Combination studies were also performed in the human NSCLC line HCC827. Interestingly, synergy is seen when the combination of the EGFR small molecule tyrosine kinase inhibitor erlotinib is used with A-928605. The effect of dual IGF1R/EGFR inhibition leads to a noticeable regression in these tumors. This is of particular interest because clinical data suggests that both the EGFR pathway and the IGF1R pathway are implicated in NSCLC as discussed below.

There are additional small molecule clinical candidates directed against IGF1R that are being investigated, but monoclonal antibodies targeting IGF1R are currently the most clinically advanced reagents. These IGF1R specific antibodies show clear inhibition of tumor growth in numerous pre-clinical tumor models [37-40]. In the clinic, these IGF1R antibodies have produced compelling proof-of-principle data that implicate an important role for IGF signaling in numerous tumor types including, but not limited to, NSCLC and Ewing's Sarcoma [35]. Based on these preliminary clinical results and the preponderance of evidence that IGF plays an essential role in cancer, it is important that development of small molecule inhibitors of IGF1R continue and that they are able to have their clinical efficacy assessed in a patient population in need of more molecularly targeted therapies such as A-928605.

## Conclusion

In this study, we demonstrated the *in vitro *and *in vivo *activities of A-928605, a novel pyrazolo [3,4-*d*]pyrimidine small molecule inhibitor of IGF1R. IGF signaling is believed to have multiple roles in cancer from oncogenesis to metastasis making it compelling for a targeted therapy approach. A-982605 is selective at inhibiting IGF1R and effective as a single agent or in combination with clinically approved agents targeting EGFR in several cancer models *in vivo*. Our results suggest that A-928605 or similar molecules it could provide useful clinical therapeutics in the treatment of cancer.

## Abbreviations

b.i.d.: twice daily; EGFR: epidermal growth factor receptor; ERK: extracellular signal regulated kinase; FDR: false discovery rate; IGF: insulin-like growth factor; IGF1R: insulin-like growth factor-1 receptor; i.p.: intraperitoneally; IPA: Ingenuity Pathways Analysis; IR: insulin receptor; NSCLC: non-small cell lung cancer; RTK: receptor tyrosine kinase; T/C: treated versus control;

## Competing interests

The authors declare that they have no competing interests. All authors are employees of Abbott Laboratories and this study was funded by Abbott Laboratories.

## Authors' contributions

WNP designed and helped perform the *in vitro *experiments and drafted the original manuscript. PMJ analyzed the microarray results and participated in drafting of the manuscript. JAM, YW, CD and FGB designed and performed the *in vivo *experiments. MMG carried out the microarray experiments. RDH synthesized A-928605 and aided in experimental design. NBS and EFJ performed the kinome analysis. QZ participated in generation of cell lines and helped perform proliferation assays. JW, SKD, GSS and RLB helped conceive the study, assisted in the study design and interpretation of the data. JW supervised and coordinated the study and finalized the manuscript. All authors read and approved the manuscript.

## Pre-publication history

The pre-publication history for this paper can be accessed here:

http://www.biomedcentral.com/1471-2407/9/314/prepub

## Supplementary Material

Additional File 1 **Supplementary Figure S1. Transformed morphology of the CD8-IGF1R cells**. **A**, Phase contrast images of the NIH-3T3 vector control cells versus the NIH-3T3 CD8-IGF1R cells. **B**, Morphological dose response of the CD8-IGF1R line to A-928605 treatment. Cells were plated and allowed to adhere and proliferate overnight. The next day compound was added and imaging was performed two days later. The cellular morphology appears to revert back towards a naive 3T3 fibroblast at concentrations near or below the IC_50 _of A-928605. All images were captured with a 20× objective. Click here for file

Additional File 2 **Supplementary Figure S2. A-928605 inhibits growth of CD8-IGF1R cells in soft agar**. Cells were plated in soft agar with an overlay containing DMSO control (left) or a final concentration of 1 μM A-928605 (right). Cells were allowed to grow in a tissue culture incubator for three weeks before imaging. The 3T3 vector control line was not capable of anchorage-independent growth in soft agar (data not shown). Click here for file

Additional File 3 **Supplementary Table 1**. Key Affymetrix probe sets related to cell cycle, growth, proliferation and IGF signaling with significant changes (5% False Discovery Rate (FDR), fold change > 1.5) in any of the following three comparisons: vehicle-treated CD8-IGF1R line relative to the vehicle-treated NIH-3T3 vector control line, A-928605-treated 3T3 CD8-IGF1R relative to vehicle-treated 3T3 CD8-IGF1R, or A-928605 treated NIH-3T3 vector control line relative to the vehicle-treated NIH-3T3 vector control line. Click here for file
