# Supplementary figures and images for: Reversal of oncogene transformation and suppression of tumor growth by the novel IGF1R kinase inhibitor A-928605
Source: BMC Cancer. 2009 Sep 4;9:314. doi: 10.1186/1471-2407-9-314 (PMC2749869; doi:10.1186/1471-2407-9-314)

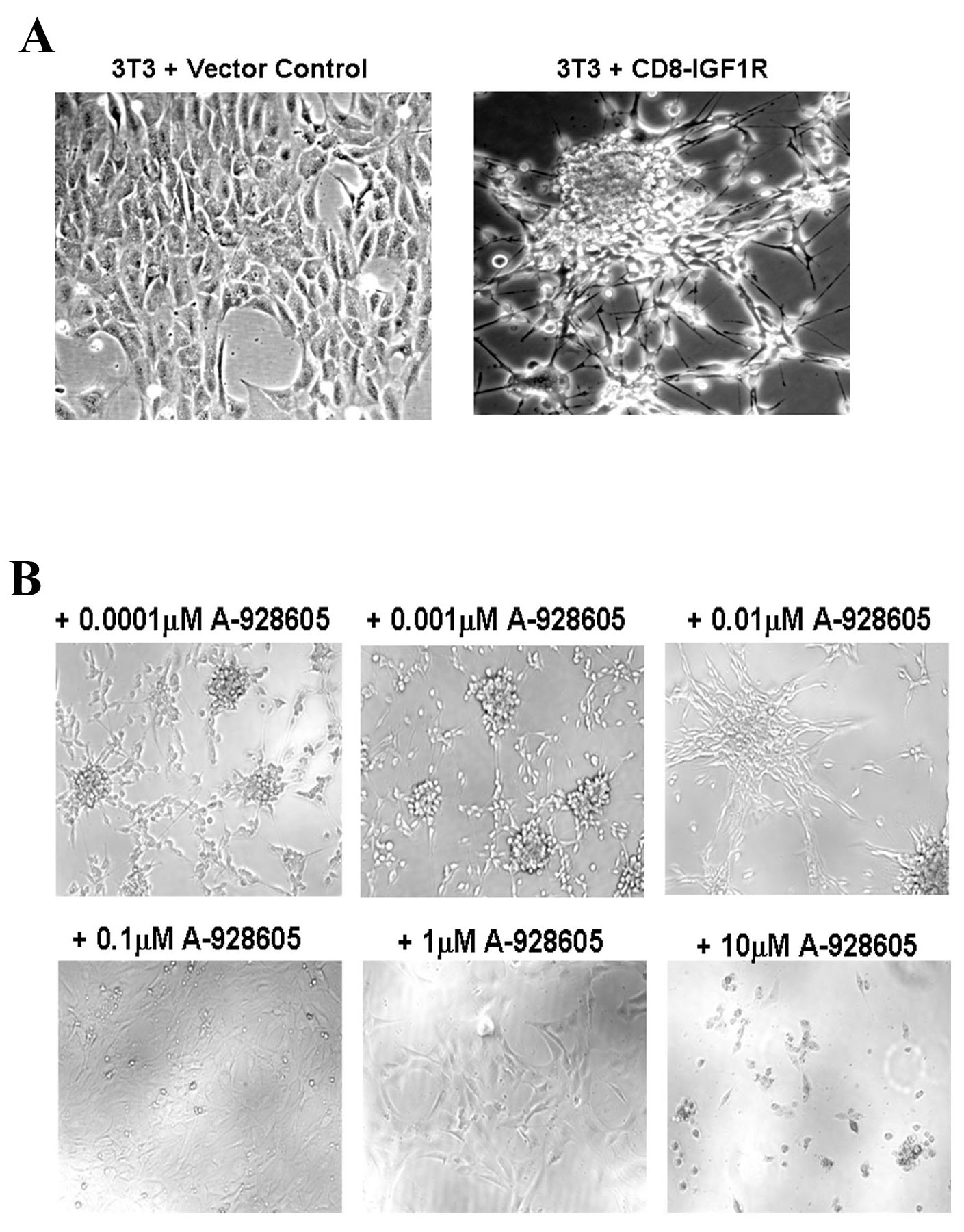

Supplement: Additional File 1 — Supplementary Figure S1. Transformed morphology of the CD8-IGF1R cells. A, Phase contrast images of the NIH-3T3 vector control cells versus the NIH-3T3 CD8-IGF1R cells. B, Morphological dose response of the CD8-IGF1R line to A-928605 treatment. Cells were plated and allowed to adhere and proliferate overnight. The next day compound was added and imaging was performed two days later. The cellular morphology appears to revert back towards a naive 3T3 fibroblast at concentrations near or below the IC50 of A-928605. All images were captured with a 20× objective. [file 1471-2407-9-314-S1.jpeg]

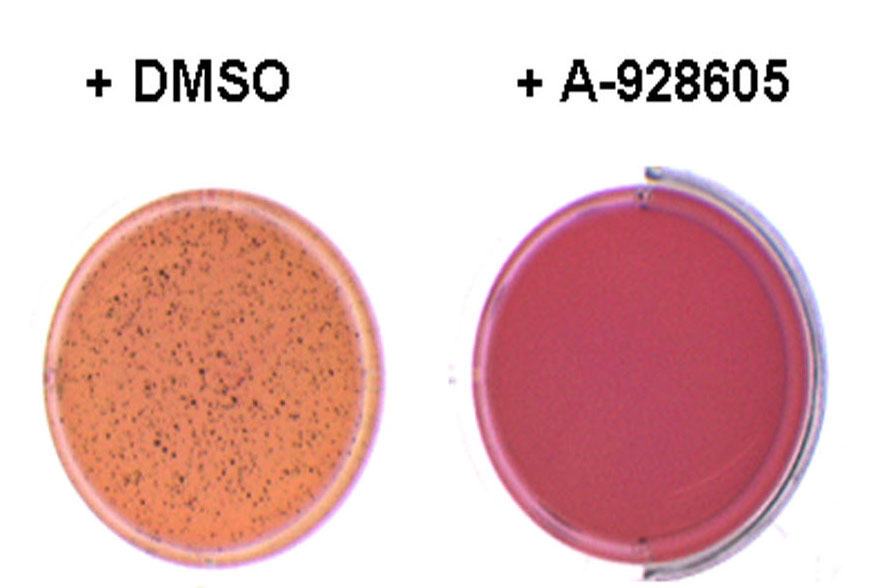

Supplement: Additional File 2 — Supplementary Figure S2. A-928605 inhibits growth of CD8-IGF1R cells in soft agar. Cells were plated in soft agar with an overlay containing DMSO control (left) or a final concentration of 1 μM A-928605 (right). Cells were allowed to grow in a tissue culture incubator for three weeks before imaging. The 3T3 vector control line was not capable of anchorage-independent growth in soft agar (data not shown). [file 1471-2407-9-314-S2.jpeg]
