# Supplementary material for: Reversal of oncogene transformation and suppression of tumor growth by the novel IGF1R kinase inhibitor A-928605
Source: BMC Cancer. 2009 Sep 4;9:314. doi: 10.1186/1471-2407-9-314 (PMC2749869; doi:10.1186/1471-2407-9-314)
Supplement: Additional File 3 — Supplementary Table 1. Key Affymetrix probe sets related to cell cycle, growth, proliferation and IGF signaling with significant changes (5% False Discovery Rate (FDR), fold change > 1.5) in any of the following three comparisons: vehicle-treated CD8-IGF1R line relative to the vehicle-treated NIH-3T3 vector control line, A-928605-treated 3T3 CD8-IGF1R relative to vehicle-treated 3T3 CD8-IGF1R, or A-928605 treated NIH-3T3 vector control line relative to the vehicle-treated NIH-3T3 vector control line. [file 1471-2407-9-314-S3.rtf]

Supplemental Table 1

		3T3 CD8-IGF1R Vehicle / 3T3 Vector Control Vehicle	3T3 CD8-IGF1R              A-928605 /  Vehicle	3T3 Vector Control         A-928605 / Vehicle	
Probe Set ID	Gene Symbol	Fold Change	P-value	Fold Change	P-value	Fold Change	P-value	
1440879_at	Abca9	-5.2	4.45E-42	1.0	0.971	-1.7	1.75E-07	
1456590_x_at	Akr1b3	1.5	5.61E-10	3.3	1.00E-45	1.7	1.66E-21	
1421134_at	Areg	36.0	2.80E-45	-32.0	9.02E-28	1.4	0.602	
1448135_at	Atf4	1.8	3.52E-08	-1.9	3.34E-06	-1.1	0.423	
1453288_at	Atf6	1.7	0.068	-3.8	4.17E-05	-1.2	0.627	
1424511_at	Aurka	2.1	4.80E-15	-9.7	1.00E-45	1.0	0.938	
1424128_x_at	Aurkb	1.5	1.94E-04	-17.0	1.00E-45	1.0	0.912	
1422169_a_at	Bdnf	1.8	1.31E-06	-1.7	3.04E-05	1.1	0.718	
1424278_a_at	Birc5	1.9	2.82E-43	-28.9	1.00E-45	-1.1	0.066	
1421161_at	Btc	3.1	0.002	-1.3	0.462	1.7	0.271	
1424046_at	Bub1	1.9	2.96E-30	-14.0	1.00E-45	-1.0	0.455	
1416961_at	Bub1b	1.9	1.70E-08	-17.1	1.00E-45	-1.3	0.144	
1417910_at	Ccna2	1.4	2.47E-13	-13.7	1.00E-45	-1.0	0.799	
1419943_s_at	Ccnb1	1.4	6.66E-05	-23.6	1.00E-45	-1.1	0.482	
1450920_at	Ccnb2	1.8	6.42E-08	-8.5	1.00E-45	1.0	0.959	
1417420_at	Ccnd1	2.5	1.00E-45	-3.1	1.00E-45	1.1	0.034	
1416122_at	Ccnd2	-1.4	5.10E-13	-1.7	1.97E-25	-1.1	0.299	
1416492_at	Ccne1	1.8	1.49E-09	-3.4	2.93E-17	-1.1	0.617	
1422535_at	Ccne2	1.2	0.262	-4.0	2.24E-15	1.0	0.765	
1422513_at	Ccnf	1.8	1.58E-06	-8.4	1.60E-33	-1.1	0.395	
1420827_a_at	Ccng1	1.1	8.96E-04	1.2	9.10E-08	1.1	0.120	
1416488_at	Ccng2	1.2	2.36E-06	1.6	6.38E-38	1.0	0.443	
1418585_at	Ccnh	-1.5	0.002	-1.6	0.002	-1.2	0.276	
1429418_at	Cdc14b	-1.2	0.025	-2.2	3.31E-17	-1.1	0.421	
1437070_at	Cdc14b	-1.0	0.535	-1.8	2.97E-08	-1.0	0.723	
1416664_at	Cdc20	1.2	0.108	-8.2	4.95E-27	1.0	0.878	
1421963_a_at	Cdc25b	1.5	1.22E-04	-2.9	1.97E-16	1.0	0.929	
1422252_a_at	Cdc25c	3.4	1.46E-08	-19.1	7.75E-23	-1.2	0.544	
1456077_x_at	Cdc25c	3.3	7.56E-12	-8.4	2.25E-15	-1.1	0.730	
1448314_at	Cdc2a	1.2	0.003	-6.4	1.00E-45	-1.1	0.376	
1434997_at	Cdc2l6	-1.0	0.796	1.5	1.50E-08	1.0	0.816	
1435807_at	Cdc42	7.3	3.08E-20	-2.3	2.17E-06	-1.3	0.469	
1424376_at	Cdc42ep1	1.1	0.088	2.2	1.00E-45	-1.1	0.046	
1416575_at	Cdc45l	2.8	1.86E-13	-2.4	5.75E-11	-1.0	0.995	
1417019_a_at	Cdc6	2.0	1.57E-10	-23.3	1.00E-45	-1.0	0.952	
1426002_a_at	Cdc7	1.5	7.55E-14	-2.8	1.00E-45	-1.1	0.250	
1443986_at	Cdc73	1.6	0.032	-2.2	0.002	-1.0	0.932	
1437251_at	Cdca2	1.7	3.02E-06	-15.3	2.75E-42	-1.1	0.383	
1452040_a_at	Cdca3	1.3	1.87E-09	-6.9	1.00E-45	-1.0	0.862	
1423683_at	Cdca4	1.3	0.054	-2.8	5.60E-06	-1.0	0.993	
1448466_at	Cdca5	1.7	9.42E-06	-4.5	4.15E-24	1.1	0.706	
1428480_at	Cdca8	1.5	0.120	-16.1	4.58E-12	1.1	0.828	
1447617_at	Cdk2	-2.9	0.004	1.2	0.737	-3.1	1.90E-04	
1424638_at	Cdkn1a	1.6	1.56E-09	1.5	1.85E-08	-1.1	0.191	
1434045_at	Cdkn1b	-1.5	1.68E-09	1.4	1.83E-07	1.2	0.019	
1430574_at	Cdkn3	-1.0	0.874	-2.3	6.99E-09	1.1	0.379	
1450677_at	Chek1	1.2	0.193	-7.5	4.19E-25	1.0	0.850	
1422747_at	Chek2	2.2	1.00E-45	-4.0	1.00E-45	1.0	0.961	
1455393_at	Cp	3.1	1.00E-45	-1.3	1.21E-05	-1.6	2.11E-05	
1441855_x_at	Cxcl1	1.9	7.60E-18	1.6	2.91E-11	1.1	0.251	
1417574_at	Cxcl12	-26.3	1.00E-45	1.9	7.94E-08	-1.0	0.593	
1419728_at	Cxcl5	-1.8	1.08E-15	2.7	1.00E-45	-1.1	0.506	
1418334_at	Dbf4	2.5	4.08E-13	-2.2	7.50E-16	1.1	0.449	
1420636_a_at	Dusp12	-1.4	1.30E-09	-1.4	7.11E-06	-1.0	0.715	
1428834_at	Dusp4	3.0	1.25E-37	-2.9	3.91E-42	1.1	0.600	
1415834_at	Dusp6	5.1	1.00E-45	-9.2	1.00E-45	1.0	0.586	
1454737_at	Dusp9	2.5	9.04E-06	-2.1	3.93E-05	1.3	0.326	
1417878_at	E2f1	3.2	1.02E-04	-2.3	0.019	-1.2	0.666	
1455790_at	E2f2	2.9	1.16E-04	-10.6	2.34E-08	-1.4	0.370	
1451924_a_at	Edn1	-72.4	1.00E-45	6.8	3.33E-04	1.1	0.312	
1433525_at	Ednra	5.6	1.43E-20	-6.4	4.16E-32	-1.9	0.065	
1435888_at	Egfr	-4.1	1.98E-42	-1.9	1.28E-04	-1.3	0.004	
1417065_at	Egr1	1.4	1.06E-08	-1.2	7.18E-05	-1.8	7.97E-29	
1424919_at	Erbb2	-1.8	0.011	-1.1	0.749	-1.1	0.658	
1419431_at	Ereg	9.6	1.00E-45	-5.1	1.00E-45	1.1	0.654	
1420847_a_at	Fgfr2	-1.5	0.002	1.5	0.005	1.1	0.598	
1438953_at	Figf	-6.4	1.00E-45	2.0	5.19E-28	1.2	1.43E-04	
1423100_at	Fos	-8.9	9.88E-09	1.0	0.968	-4.3	1.35E-05	
1419080_at	Gdnf	4.8	9.41E-18	-1.9	1.45E-06	1.1	0.757	
1451501_a_at	Ghr	-3.1	1.00E-45	1.8	1.15E-36	-1.1	0.030	
1423804_a_at	Idi1	1.5	1.75E-05	-4.5	1.90E-38	1.7	7.02E-05	
1426565_at	Igf1r	-1.8	2.33E-06	1.0	0.84	-1.0	0.767	
1448152_at	Igf2	-20.5	1.00E-45	3.9	2.36E-06	-1.3	0.010	
1437405_a_at	Igfbp4	6.6	1.00E-45	1.5	2.96E-09	1.2	0.166	
1452114_s_at	Igfbp5	-4.2	2.00E-04	1.3	0.666	-1.1	0.694	
1426207_at	Ikbkb	-2.0	4.44E-35	1.3	1.21E-06	-1.0	0.576	
1435646_at	Ikbkg	-1.3	0.006	2.4	1.08E-20	1.1	0.586	
1449982_at	Il11	2.6	4.26E-06	-2.3	1.33E-04	-1.3	0.430	
1454671_at	Insig1	1.1	0.005	-1.7	2.45E-43	1.2	4.09E-07	
1417982_at	Insig2	1.6	1.42E-22	1.1	0.014	-1.1	0.159	
1423104_at	Irs1	-2.8	1.00E-45	2.3	1.00E-45	-1.0	0.099	
1443969_at	Irs2	-4.6	1.00E-45	1.7	5.88E-04	-1.0	0.778	
1421207_at	Lif	8.7	1.00E-45	-1.6	2.30E-05	-1.1	0.700	
1420013_s_at	Lss	1.6	4.65E-12	-4.9	1.00E-45	1.5	7.50E-08	
1451714_a_at	Map2k3	1.0	0.873	-1.6	2.05E-14	1.0	0.481	
1426850_a_at	Map2k6	1.8	5.58E-04	-2.2	7.39E-04	1.2	0.300	
1421877_at	Mapk9	1.2	8.35E-05	-1.3	3.43E-11	-1.0	0.620	
1427718_a_at	Mdm2	1.5	0.002	-1.8	2.35E-04	-1.0	0.783	
1417656_at	Mybl2	1.3	0.167	-2.6	1.81E-05	1.3	0.112	
1424942_a_at	Myc	1.3	0.003	-1.9	4.45E-14	1.3	0.008	
1429128_x_at	Nfkb2	-1.2	0.068	1.6	1.91E-05	-1.0	0.711	
1419675_at	Ngfb	3.1	2.16E-13	-2.5	3.54E-09	-1.5	0.109	
1416222_at	Nsdhl	1.3	0.004	-2.3	6.15E-28	1.5	5.31E-07	
1419663_at	Ogn	-12.8	1.00E-45	3.8	1.00E-45	1.1	0.254	
1417947_at	Pcna	1.1	0.012	-2.2	1.00E-45	-1.1	0.005	
1460326_at	Pik3ca	-1.3	5.03E-08	1.4	9.78E-15	-1.1	0.001	
1438682_at	Pik3r1	-1.9	1.00E-45	1.1	0.004	-1.0	0.156	
1456482_at	Pik3r3	-1.7	2.52E-06	1.7	2.63E-06	-1.2	0.104	
1448191_at	Plk1	2.0	2.49E-23	-9.8	1.00E-45	-1.1	0.351	
1434496_at	Plk3	6.3	2.86E-06	-7.8	5.76E-05	-1.2	0.782	
1423059_at	Ptk2	2.1	5.61E-20	-1.9	1.58E-13	1.0	0.678	
1424156_at	Rbl1	1.4	2.06E-12	-5.8	1.00E-45	-1.0	0.426	
1425166_at	Rbl1	3.4	1.89E-04	-7.7	3.08E-07	-1.1	0.882	
1419536_a_at	Rela	-1.2	0.145	1.6	2.87E-05	1.1	0.441	
1451960_a_at	Rev3l	-1.0	0.982	1.4	0.234	-5.7	4.71E-06	
1418969_at	Skp2	1.2	0.001	-2.6	1.00E-45	1.0	0.527	
1448377_at	Slpi	4.6	1.00E-45	1.1	0.164	-1.7	8.92E-10	
1420895_at	Tgfbr1	1.6	8.59E-27	-1.5	1.57E-18	-1.1	0.278	
1419607_at	Tnf	1.1	0.800	2.6	0.003	1.3	0.584	
1449033_at	Tnfrsf11b	2.2	3.42E-04	-2.1	5.78E-04	1.2	0.506	
1418571_at	Tnfrsf12a	1.9	1.28E-05	-2.7	3.59E-11	-1.1	0.74	
1422038_a_at	Tnfrsf22	4.3	6.39E-13	-1.6	0.005	-1.0	0.971	
1420351_at	Tnfrsf4	2.7	2.24E-04	-2.2	0.002	1.3	0.409	
1418175_at	Vdr	-2.2	2.70E-08	2.1	1.27E-10	-1.1	0.354	
1420909_at	Vegfa	4.3	1.00E-45	1.0	0.963	1.1	0.489	
1419417_at	Vegfc	-7.4	1.00E-45	1.1	0.606	-1.1	0.500	
